# Using trauma registry data to predict prolonged mechanical ventilation in patients with traumatic brain injury: Machine learning approach

**DOI:** 10.1371/journal.pone.0235231

**Published:** 2020-07-08

**Authors:** Ahmad Abujaber, Adam Fadlalla, Diala Gammoh, Husham Abdelrahman, Monira Mollazehi, Ayman El-Menyar

**Affiliations:** 1 Assistant Executive Director of Nursing, Hamad medical corporation, Doha, Qatar; 2 Management Information Systems, Business, and Economics faculty at Qatar University, Doha, Qatar; 3 Industrial Engineering, University of Central Florida, Orlando, Florida, United States of America; 4 Department of Surgery, Trauma Surgery, Hamad Medical Corporation, Doha, Qatar; 5 Department of Surgery, Trauma Surgery, Clinical Research, Hamad Medical Corporation, Doha, Qatar; 6 Department of Clinical Medicine, Weill Cornell Medical college, Doha, Qatar; University Hospital Zurich, SWITZERLAND

## Abstract

**Objectives:**

We aimed to build a machine learning predictive model to predict the risk of prolonged mechanical ventilation (PMV) for patients with Traumatic Brain Injury (TBI).

**Methods:**

This study included TBI patients who were hospitalized in a level 1 trauma center between January 2014 and February 2019. Data were analyzed for all adult patients who received mechanical ventilation following TBI with abbreviated injury severity (AIS) score for the head region of ≥ 3. This study designed three sets of machine learning models: set A defined PMV to be greater than 7 days, set B (PMV > 10 days) and set C (PMV >14 days) to determine the optimal model for deployment. Patients’ demographics, injury characteristics and CT findings were used as predictors. Logistic regression (LR), Artificial neural networks (ANN) Support vector machines (SVM), Random Forest (RF) and C.5 Decision Tree (C.5 DT) were used to predict the PMV.

**Results:**

The number of eligible patients that were included in the study were 674, 643 and 622 patients in sets A, B and C respectively. In set A, LR achieved the optimal performance with accuracy 0.75 and Area under the curve (AUC) 0.83. SVM achieved the optimal performance among other models in sets B with accuracy/AUC of 0.79/0.84 respectively. ANNs achieved the optimal performance in set C with accuracy/AUC of 0.76/0.72 respectively. Machine learning models in set B demonstrated more stable performance with higher prediction success and discrimination power.

**Conclusion:**

This study not only provides evidence that machine learning methods outperform the traditional multivariate analytical methods, but also provides a perspective to reach a consensual definition of PMV.

## Background

Patients with severe traumatic brain injury (TBI) are prone to impaired arousal which warrants protecting their airway by mechanical ventilation (MV) [1]. Therefore, they are at a higher risk of prolonged mechanical ventilation (PMV) than any critical patients [2]. In 2007, the European Respiratory Journal published the weaning from mechanical ventilation guidelines to describe the entire process of liberating patients from the ventilator [3]. Nonetheless, due to the lack of robust evidence in the literature, there were no clear recommendations about the weaning process in the neurocritical care settings which made the decision to extubate the patient a complex decision [2].

Although MV is a lifesaving intervention, it has several complications such as ventilator- induced lung injury, ventilator associated pneumonia (VAP), prolonged hospitalization and mortality [4, 5]. These risks increase with the PMV [5, 6]. Approximately, 30% of critically ill patients requires PMV [5, 7, 8]. It is predicted that more than 600,000 patients per year will require PMV in 2020 [9]. Several strategies, such as minimizing the sedation and performing daily spontaneous breathing trials have been adopted to mitigate the risks associated with the MV and to prevent the PMV [10, 11].

Hence, predicting patients at risk for PMV is of utmost importance to help clinicians design individualized plans of care that mitigate the risk of PMV. This includes the decision of early use of tracheostomy which has been proven beneficial when MV is still required [8, 12–14]. There are several studies that aimed to determine the significant predictors of PMV. However, it remains difficult to determine a set of key predictors due to the differences in patients’ clinical features and clinical settings. Furthermore, there is no consensus on the definition of PMV. The PMV period in the published literature ranges from 5 hours to 1 year with > 21 days being the most common definition for PMV [15]. **Table 1** shows examples of the previously published literature in predicting PMV highlighting the patients’ characteristics, PMV duration, used predictors and the predictive models’ performance measures.

**Table 1:** Examples of past literature on predicting PMV.

Study	Patient group	PMV duration	Predictors	Predictive technique	Model`s performance (AUC)
Parreco et al. (2018) [8]	All ventilated level 3 ICU patients (2001–2012)	>7days	Oxford Acute Severity of Illness Score (OAISIS), Sequential Organ Failure Assessment, Simplified Acute Physiology Score (SAPS), Simplified Acute Physiology Score II (SAPS II), Acute Physiology Score III, and logistic organ dysfunction score (LODS), Sepsis Related Organ Failure Assessment (SOFA)	Gradient-Boosted Decision Tree Algorithm	Mean AUC 0.820 ± 0.016
Chang et al (2018) [16]	ICU patients who survived the Sepsis/Septic shock and respiratory failure	>21 days	Demographics Acute Physiology, Age, Chronic Health Evaluation (APACHE II) Comorbidities Lab findings (hematology, liver function, coagulation, Urea Electrolytes, Arterial blood gases) Ventilator settings	Logistic Regression	AUC 0.725
Agle et al. (2006) [12]	Torso trauma patients who met specific criteria for shock resuscitation and required 48 hours of mechanical ventilation	>14 days	Demographics, Facial trauma, chest trauma severity (abbreviated injury score AIS), ventilatory settings.	Logistic Regression	AUC 0.79
Clark and Lettieri (2013) [5]	Adult patients requiring MV support in a medical intensive care unit (ICU)	>14 days	Demographics, vital signs, laboratory values (hematology, renal and liver function tests, HCO3), APACHE 2	Logistic regression	AUC 0.75
Dimopoulou et al. (2003) [13]	Adult patients with thoracic trauma requiring MV support in a intensive care unit (ICU)	>7 days	Demographics, injury characteristics, Injury severity score, AIS of the other associated injuries (head, neck, face, pelvis and extremities) and ventilatory settings	Logistic regression	Not declared
Figueroa- Casas et al. (2015) [14]	ICU patients receiving MV	>7 days	Demographics, SOFA score on intubation, comorbidities, location before ICU admission, diagnosis category	Logistic regression	AUC 0.65–0.70

In a recent Cochrane systematic review, the early tracheostomy (<10 days from the start of MV) was found to be associated with significant improvement of patient’s treatment outcomes [17]. This finding supports the previous randomized clinical trial by Young et al. who found that early tracheostomy replacement (< 10 days) is beneficial to the patients and is associated with improved outcomes [18]. Besides the favorable clinical outcomes, early tracheostomy is associated with improved economic outcomes such as reduced intensive care unit (ICU) cost [19] and reduced hospital length of stay [17]. Furthermore, early tracheostomy was found to significantly improve the patient`s quality of life (QOL) compared to the endotracheal ventilation when prolonged ventilation is required [20]. Therefore, defining the PMV to be longer than 10 days could be of a great value if early liberation from MV, early tracheostomy replacement, improving quality of life and cost-effectiveness were concerned. Most of the previously published studies that aimed to predict PMV, used the conventional multivariate techniques particularly logistic regression and yielded low to moderate accuracies (0.53–0.75) and Area under the curve (AUC) between 0.65and 0.75 [8, 14]. The implementation of the machine learning to predict the PMV has achieved a relatively higher performance than the conventional predictive models with accuracy of 0.832 and AUC of 0.82 [8]. Accordingly, we decided in this study to evaluate the predictive performance of selected machine learning models when PMV is defined as > 10 days. At the same time, we conducted another two sets of predictive models in which PMV is defined as > 7days and > 14 days in order to compare the predictive performance of the machine learning models in the three sets.

### Methodology

This study utilized the trauma registry data to design supervised machine learning algorithms to predict the PMV (> 7 days, > 10 days and > 14 days) for patients who received MV following moderate to severe TBI. We hypothesized that the machine learning algorithms outperform the conventional multivariate predictive techniques in terms of accuracy, sensitivity, specificity, and precision, Negative Predictive Value (NPV), F-score and AUC. Also, consistent with the Cochrane`s systematic review results, we hypothesized that defining PMV to be greater than 10 days might help optimize the prediction performance of the machine learning models used in this study.

**Fig 1:**
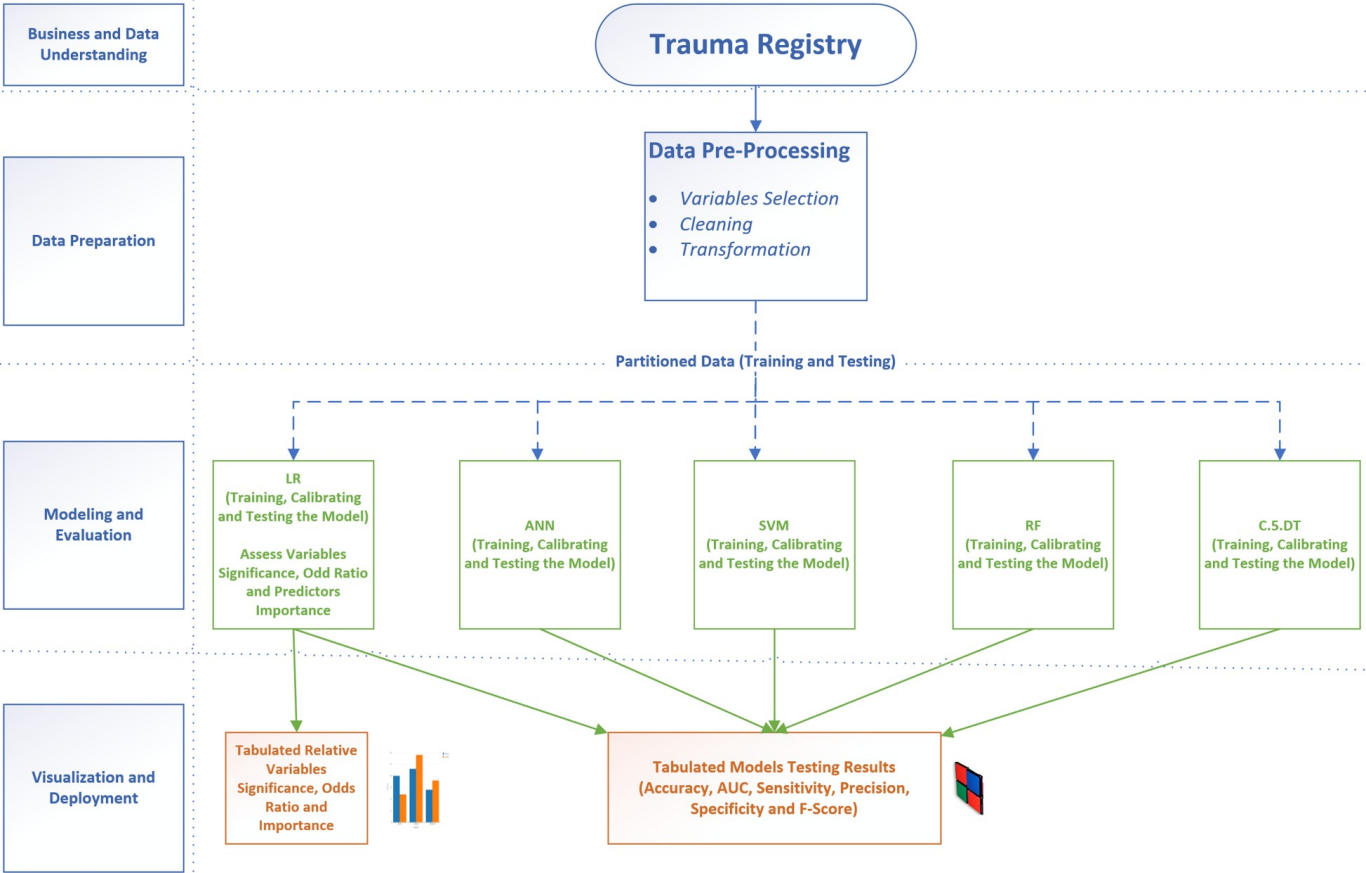
Research methodology.

The study was conducted in accordance with the Cross-Industry Standard Process for Data Mining (CRISP-DM) that provides a definition of typical phases of the data mining projects. CRISP-DM breaks the data mining process into six phases: business and data understanding, data preparation, modeling, evaluation and deployment [21]. **Fig 1** summarizes the methodology that was followed in this study.

We constructed three sets of predictive models based on the definition of the PMV. Set A defines PMV as > 7 days, set B defines PMV as > 10 days and set C defines PMV as >14 days.

#### Business and data understanding

Not all the registry data were usable in this study. Therefore, to better understand and choose meaningful variables, we explored the definition of each variable in the trauma registry data dictionary. In addition, we reviewed the literature in order to determine which among the enormous number of variables need to be considered a predictor and which among them to be imputed if in case they have missing values [22]. Head Abbreviated Injury Scale Score (HAIS) was used to classify the severity of TBI. Head AIS of 3 and 4 were considered moderate severity and HAIS 5 was considered severe TBI [23, 24].

#### Data preparation

The study was approved by the Institutional Review Board (IRB) of Hamad Medical Corp. (HMC) in Qatar. This retrospective study targeted all adult patients who were admitted to level 1 trauma center at Hamad General Hospital (HGH) in the period from January 2014 to February 2019 and registered in the trauma registry (a prospectively collected standardized and well maintained data with robust quality assurance). A total of 2318 patients with TBI were registered in the trauma registry for the given period.

The following inclusion criteria were followed to select the patient`s record:

- Adult patients older than 14 years with TBI.
- Patients whose Abbreviated Injury Score for head region (HAIS) ≥ 3
- Patients who underwent intubation following the injury either at the scene by the ambulance crew or in the hospital within the first 24 hours from the arrival to the hospital.
- On the other hand, the exclusion criteria were:
- Patients with any regional injuries that have AIS greater than the HAIS to ensure that the TBI assumes the highest effects on the dependent variable.
- All patients who died or discharged within 7 days for set A, within 10 days for set B and within 14 days for set C.

**Fig 2:**
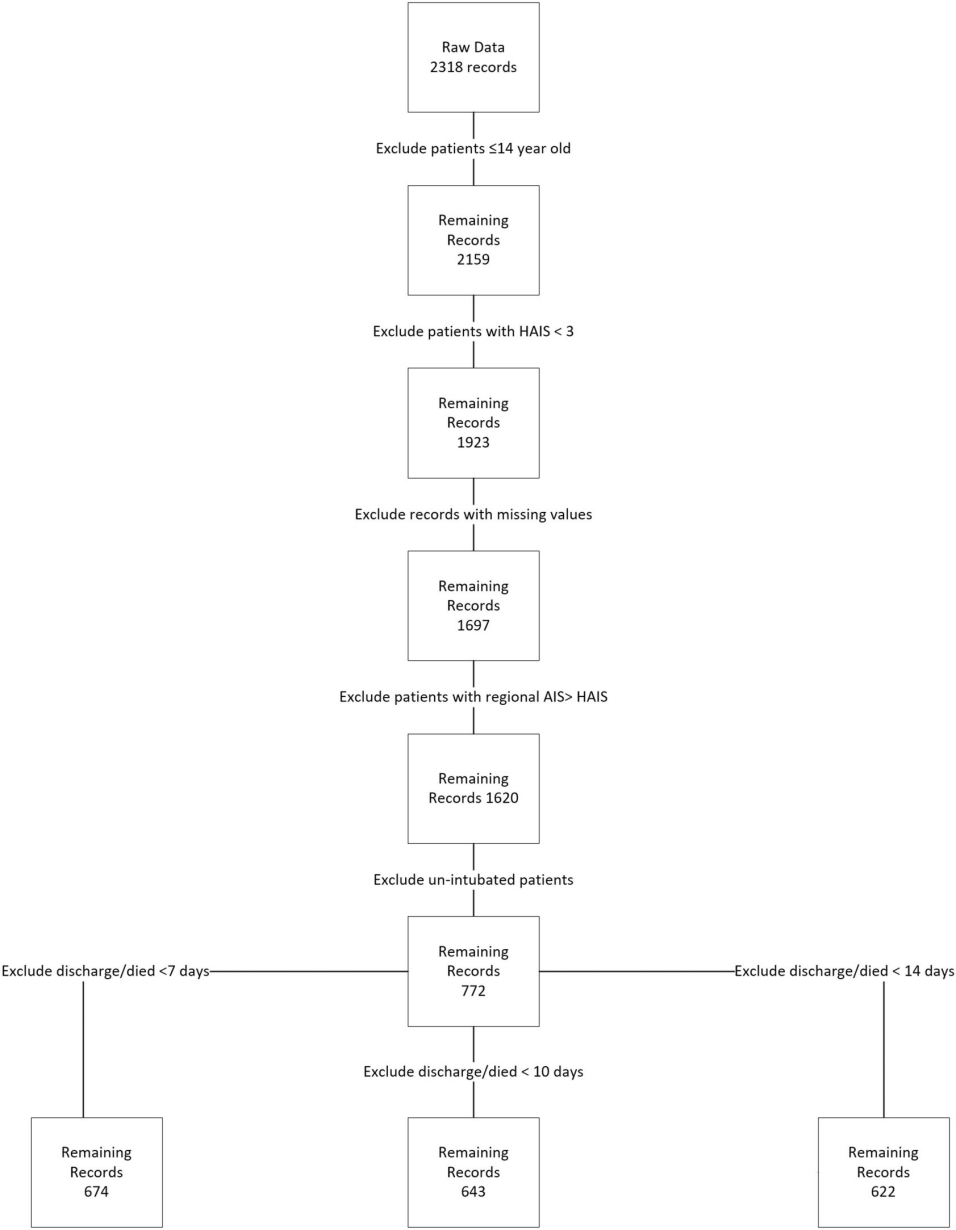
Records inclusion and exclusion procedure.

Due to the criticality of the subject, we decided that all records with missing values to be deleted. Therefore, no imputation was needed. All variables that have no predictive power (e.g. health record number, date of admission and date of disposition) or those that were severely imbalanced (e.g. gender: where female patients were approximately 4% in the three data sets) were excluded. Subsequently, 674 records in set A, 643 records in set B, and 622 records in set C were eligible for the study. Fig 2 explains the records inclusion and exclusion procedure.

The retrieved data included the following variables: age, gender, race, mechanism of injury, vital signs upon arrivals (blood pressure and heart rate) Glasgow Coma Score (GCS) on arrival to the emergency department (ED), CT scan findings, injury severity score (ISS), the AIS per body region, intubation status and location, date/time of injury, time of admission to the ED, patients known comorbidities, performed procedures, blood transfusion, in-hospital complications, outcome and date of disposition.

### Outcome measure

The dichotomous outcome measure for this study is the prolonged mechanical ventilation (PMV). PMV is defined as the stay on mechanical ventilatory support for > 7 days in set A, > 10 days in set B and > 14 days in set C from the initial intubation that was performed within the first 24 hours from the injury. PMV0 means that the patient was extubated before the sets’ period and PMV1 means that the patient stayed on MV longer than the sets’ period.

### Prediction models

A group of supervised machine learning techniques were utilized to help us to compare their performance with each other and with previous studies in order to recommend the model that achieves the optimal performance and highest practicality in supporting the clinical decision. Logistic regression (LR), Random Forrest (RF) Artificial neural networks (ANNs), C.5 Decision Tree (C.5 DT) and Support vector machines (SVMs) were selected to provide base line comparative performance. SPSS modeler 18.2 was used to conduct the analysis.

To prevent overfitting and to validate the models’ performance, we partitioned the data into training set (70%) and testing set (30%) and the overfit prevention was set at 30%. The data partitioning was executed automatically by the analytical software based on the partition command that we provided. Table 2 explains the data partitions.

**Table 2:** Data partitions.

**Set A PMV >7 days**	**Set**	**Proportion**	**Number of cases**	**Ventilator days ≤ 7**	**Ventilator days > 7**
	**Training set**	70%	472	183	289
	**Testing set**	30%	202	87	115
	**Total**	**100%**	**674**	**270**	**404**
**Set B PMV >10 days**	**Set**	**Proportion**	**Number of cases**	**Ventilator days ≤ 10**	**Ventilator days > 10**
	**Training set**	70%	446	239	207
	**Testing set**	30%	197	114	83
	**Total**	**100%**	**643**	**353**	**290**
**Set C PMV >14 days**	**Set**	**Proportion**	**Number of cases**	**Ventilator days ≤ 14**	**Ventilator days > 14**
	**Training set**	70%	432	312	120
	**Testing set**	30%	190	138	52
	**Total**	**100%**	**622**	**450**	**172**

#### Logistic Regression (LR)

LR is a typical technique for predicting binary, binomial or multinomial outcomes [22]. Step-wise LR was used in this study to control the effect of confounding variables and to measure the independent risk factors for post-TBI PMV [25].

#### Random Forrest (RF)

RF is a powerful supervised machine learning technique that is used widely for classification problems [22, 26]. RF is proven to have improved accuracy in comparison to other machine learning techniques. The reason is that RF uses bootstrapping to grow a forest of uncorrelated trees with a high degree of randomness in feature selection which contributes to reducing errors significantly [27].

#### Support Vector Machines (SVM)

SVM is a powerful classification machine learning algorithm that can be used for linear and non-linear data sets [28]. When using SVM for classification purpose, it is very important to decide which kernel function better achieves the optimal hyperplane that separates the classes [29]. Linear kernel was used in this study as it provided a better predictive performance in the preliminary assessment.

#### Artificial Neural Networks (ANN)

ANNs are widely used machine learning techniques that perform powerfully in classification and pattern identification [28]. Scholars consider ANNs a black-box analytical model. Nonetheless, their great potentials in supporting clinical practice through the engagement with the evidence-based medicine are undeniable [30]. Multi-layer perception (MLP) ANN is used for this study as it outperformed the Radial Basis Function (RBF) in the preliminary analysis. The typical MLP network consists of an input layer that consists of the input nodes or predictors, and an output layer that consists of neurons. It also has one or more layer of neurons that are usually called hidden layers as they are inaccessible [31]. The neural network is usually optimized by partitioning the data into separate training and test data sets which help prevent the overfitting. The training continues until the error is no further reducible [32] when used for classification, ANNs is seen as a set of connected input/output units in which each connection has a weight associated with it. This weight represents the strength of the connection between the units [33].

#### C.5 Decision Tree (DT)

C. 5 DT is the successor of the C. 4.5 DT classification data mining algorithm. Decision tree is a “classification algorithm in which each non-leaf node indicates a test on an attribute of the input cases; each branch corresponds to an outcome of the test; and each leaf node indicates a class prediction” [22]. Generally, DTs are powerful, logical and easy to interpret and to understand classification algorithms [34].

## Results

In set A (PMV> 7days), 674 eligible cases were included. Of them, 404 (59.9%) have ventilator days greater than 7 days. The overall mean age was 32.3 years and the mean age for patients with PMV was 33.6. Fifty-three percent of the patients sustained chest trauma. Of them, 61.7%, 24% and 46.5% sustained lung contusion, hemothorax and pneumothorax, respectively. The most common TBI diagnosis was subdural hemorrhage (SDH) (25%) and more than 30% of them developed midline shift on the computed tomography head images.

In set B (PMV >10 days), 643 eligible cases were included. Of them, 290 (45%) have ventilator days greater than 10 days. The overall mean age was 32.1 years and the mean age of patients with PMV was 33.6 years. Almost half of the patients sustained chest trauma. Of them, 63%, 22% and 43% sustained lung contusion, hemothorax and pneumothorax, respectively. SDH was the most common TBI diagnosis (25%) and 29% of them had midline shift. **Tables 3** and **4** show the sample characteristics in set B.

**Table 3:** Sample characteristics-continuous variables.

Model	Set B (PMV >10 days)			
Variable	N	Mean	SD	Mean when PMV >10
**Age**	643	32.1	12.9	33.6
**ISS**	643	26.8	9.5	29.5
**ED SBP**	643	128.2	26.1	126.6
**ED HR**	643	101.8	24.6	103.4

**Table 4:** Sample characteristics (set B)—nominal and ordinal variables.

Variable	Category	Count/%	PMV = 0	PMV = 1
**Race**	Asian	365/56.8%	218/59.7%	147/40.3%
	Other	278/43.2%	135/48.6%	143/51.4%
**Mechanism of Injury (MOI)**	MVC	247/38.4%	128/51.8%	119/48.2%
	Fall	159/24.7%	100/62.9%	59/37.1%
	Pedestrian	125/19.4%	59/47.2%	66/52.8%
	Other	112/17.4%	66/58.9%	46/41.1%
**Multiple rib fractures**	No	497/77.3%	294/59.2%	203/40.8%
	Yes	146/22.7%	59/40.4%	87/59.6%
**Lung contusion**	No	427/66.4%	246/57.6%	181/42.4%
	Yes	216/33.6%	107/49.5%	109/50.5%
**Hemothorax**	No	567/88.2%	325/57.3%	242/42.7%
	Yes	76/11.8%	28/36.8%	48/63.2%
**Pneumothorax**	No	495/77%	297/60%	198/40%
	Yes	148/23%	56/37.8%	92/62.2%
**Midline shift**	No	456/70.9%	264/57.9%	192/42.1%
	Yes	187/29.9%	89/47.6%	98/52.4%
**TBI diagnosis/ CT findings**	Sub-Dural Hemorrhage (SDH)	162/25.2%	89/54.9%	73/45.1%
	Extra-Dural Hemorrhage (EDH)	141/21.9%	100/70.9%	41/29.1%
	Sub-Arachnoid Hemorrhage (SAH)	53/8.2%	24/45.3%	29/54.7%
	Brain Contusion	93/14.5%	63/67.7%	30/32.3%
	Diffuse Axonal Injury (DAI)	93/14.5%	28/30.1%	65/69.9%
	Other	56/8.7	34/60.7%	22/39.3%
	Cerebral Edema	45/7%	15/33.3%	30/66.7%
**Head AIS (HAIS)**	3	225/35%	153/68%	72/32%
	4	157/24.4%	86/54.8%	71/45.2%
	5	281/40.6%	114/34.2%	147/65.8%
**Face AIS (FAIS)**	0	313/48.7%	169/54%	144/46%
	1	74/11.5%	36/48.6%	38/51.4%
	2 (AIS 3–5)	256/39.8%	148/57.8%	108/42.2%
**Chest AIS (CAIS)**	0	302/47%	197/65.2%	105/34.8%
	1 (AIS 1–2)	100/15.5%	42/42%	58/58%
	2 (AIS 3–5)	241/37.5%	114/47.3%	127/52.7%
**Abdomen AIS (AAIS)**	0	515/80.1%	301/58.4%	214/41.6%
	1 (AIS 1–5)	128/19.9%	52/40.6%	76/59.4%
**Spine AIS (SAIS)**	0	443/68.9%	262/59.1%	181/40.9%
	1 (AIS 1–5)	200/31.1%	91/45.5%	109/54.5%
**Glasgow Coma Score (GCS) category**	13–15	85/13.2%	56/65.9%	29/34.1%
	9–12	72/11.2%	53/73.6%	19/26.4%
	≤ 8	486/75.6%	244/50.2%	242/49.8%
**Known comorbidities**	No	537/83.5%	311/57.9%	226/42.1%
	Yes	106/16.5%	42/39.6%	64/60.4%
**Intubation location**	In-hospital	231/35.9%	142/61.5%	89/38.5%
	Pre-hospital	412/64.1%	211/51.2%	201/48.8%
**Blood transfusion**	No	235/36.6%	189/80.4%	46/19.6%
	Yes	408/63.5%	164/40.2%	244/59.8%
**Ventilator Associated Pneumonia (VAP)**	No	478/74.3%	311/65%	167/35%
	Yes	165/25.7%	42/25.5%	123/74.5%
**Sepsis**	No	583/90.7%	343/58.8%	240/41.2%
	Yes	60/9.3%	10/16.7%	50/83.3%
**Total**		643/100%	353/54.9%	290/45.1%

In set C (PMV >14 days), 622 eligible cases were included. Of them, 172 (28.5%) have ventilator days greater than 10 days. The overall mean age was 32 years and the mean age for patients with PMV was 33.9 years. There were 329 patients (52.9%) sustained chest trauma. Of them, 64% suffered lung contusion, 22.8% had hemothorax and 42% had pneumothorax. One quarter of the patients sustained SDH and 28.8% of them had midline shift.

### Performance of the data mining techniques

**Table 5** shows the performance evaluation metrics for the five machine learning techniques in the test data partition. All models achieved moderate accuracy (0.66–0.79). Nevertheless, since accuracy alone is insufficient measure to evaluate the overall model`s performance, AUC, precision, NPV, sensitivity, specificity and F-score measures were taken into consideration. In set A, LR and SVM achieved relative similar performance in all performance metrics. Nonetheless, LR is the preferred model for deployment as it demonstrated higher discrimination power that is of great importance to the classification function (AUC 0.83 vs. 0.80) and due to its parsimony. LR achieves similar performance with fewer numbers of dimensions. In Set B, SVM achieved the highest performance. In set C, ANNs and SVM achieved similar performance. ANNs is the preferred model as it gives higher accuracy, specificity and positive predictive power (precision).

**Table 5:** Performance of the prediction models.

**Set A (PMV > 7days)**	**Number of predictors**	**Accuracy**	**Area Under the Curve**	**Precision**	**Negative Predictive Value**	**Sensitivity**	**Specificity**	**F-Score**
**Logistic Regression**	7	0.75	0.83	0.77	0.72	0.80	0.68	0.78
**Support Vector Machine**	23	0.76	0.80	0.77	0.74	0.83	0.67	0.79
**Random Forrest**	23	0.73	0.77	0.77	0.69	0.76	0.70	0.76
**Artificial Neural Networks**	23	0.69	0.78	0.72	0.66	0.77	0.60	0.74
**C.5 Decision Tree**	19	0.66	0.65	0.70	0.60	0.70	0.61	0.70
**Set B (PMV > 10 days)**	**Number of predictors**	**Accuracy**	**Area Under the Curve**	**Precision**	**Negative Predictive Value**	**Sensitivity**	**Specificity**	**F-Score**
**Support Vector Machine**	23	0.79	0.84	0.75	0.82	0.76	0.82	0.75
**Artificial Neural Networks**	23	0.77	0.84	0.71	0.81	0.76	0.77	0.73
**Logistic Regression**	7	0.75	0.82	0.70	0.78	0.69	0.79	0.70
**Random Forrest**	23	0.75	0.80	0.67	0.84	0.81	0.71	0.73
**C.5 Decision Tree**	17	0.71	0.77	0.66	0.75	0.65	0.75	0.65
**Set C (PMV > 14 days)**	**Number of predictors**	**Accuracy**	**Area Under the Curve**	**Precision**	**Negative Predictive Value**	**Sensitivity**	**Specificity**	**F-Score**
**Artificial Neural Networks**	23	0.76	0.72	0.64	0.77	0.27	0.94	0.38
**Support Vector Machine**	23	0.74	0.74	0.54	0.77	0.29	0.91	0.38
**Logistic Regression**	6	0.73	0.75	0.52	0.77	0.29	0.90	0.37
**Random Forrest**	23	0.71	0.73	0.47	0.80	0.46	0.80	0.47
**C.5 Decision Tree**	10	0.71	0.65	0.43	0.76	0.25	0.88	0.32

Comparing the discrimination power between the three sets, set B that defines PMV to be greater than 10 days performs better than Sets A and C with AUC ranging from 0.77 to 0.84 while set A (PMV > 7days) AUC ranges from 0.65 to 0.83 and set C (PMV > 14 days) AUC ranges from 0.65 to 0.75. This implies that the discrimination power and the accuracy were more optimized when PMV was defined to be greater than 10 days.

### Prolonged mechanical ventilation predictors

**Fig 3:**
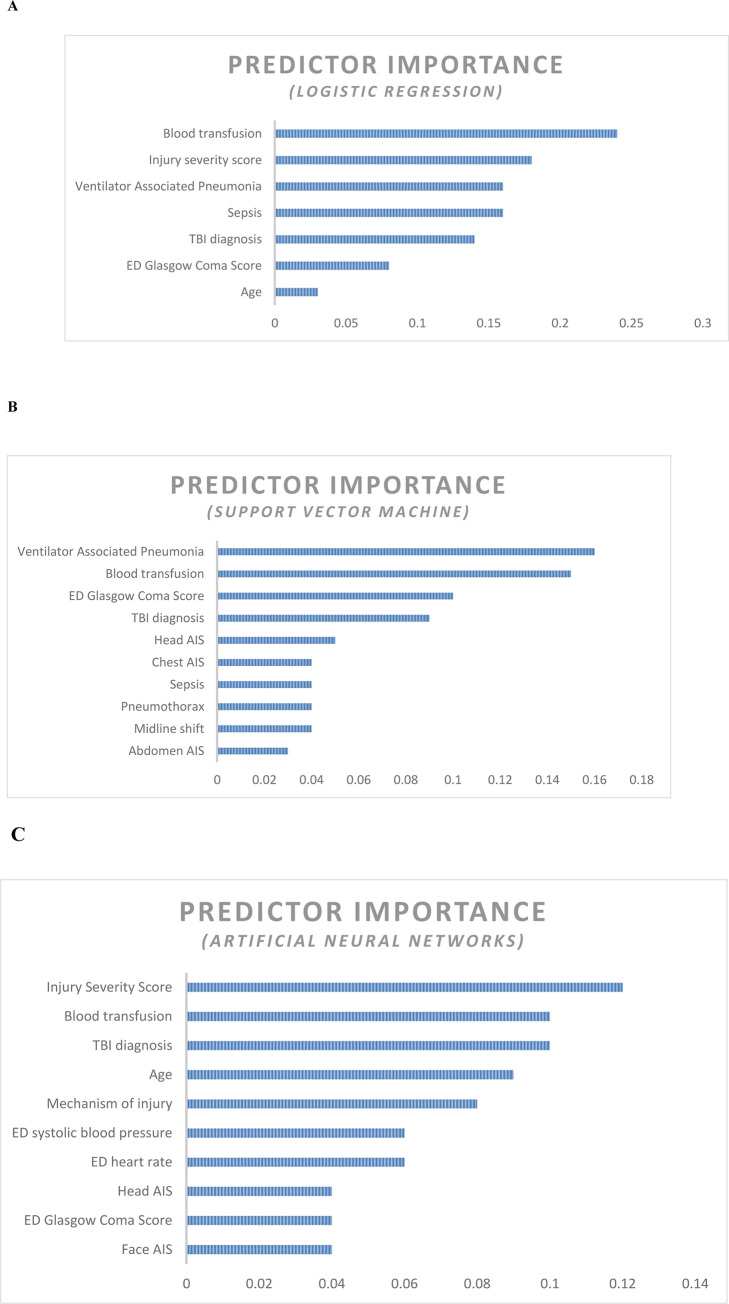
**A**. Predictor importance chart- Logistic regression (PMV> 7 days). **B**. Predictor importance chart- Support Vector Machine (PMV> 10 days). **C**. Predictor importance chart- Artificial Neural Networks (PMV> 14 days).

In set A (PMV > 7days), LR model used 7 predictors to classify the patients into two classes based on their mechanical ventilation dependency period. In machine learning, the contribution of every predictor to the reduction of error and to the overall model`s capacity to produce accurate predictions is usually presented in the form of predictor`s importance [27]. The first predictor is usually the most important predictor of the model’s capacity, then the other predictors importance values are ranked in relation to the first ranked predictor. **Fig 3A** shows the predictors importance in LR. Receiving blood during resuscitation scored the highest predictor importance value (0.24).

In set B (PMV > 10 days), the SVM used all the 27 predictors to classify the patients into the two outcome classes. Development of VAP scored the highest predictor importance value of 0.16. **Fig 3B** ranks the top ten predictors based on the predictor importance index.

In set C (PMV > 14 days), ANNs used all the 27 predictors to classify the patients into the two classes. Injury Severity Score (ISS) scored the highest predictor importance value (0.12) **Fig 3C** ranks the top ten predictors based on their importance.

Obviously, machine learning techniques in set B demonstrated more stable performance a higher discrimination power as presented by AUC. It is uncommon to define PMV to be greater than 10 days. The driver for selecting 10 days stems from the fact that the earlier liberation from MV is associated with improved patient outcomes. In addition, 10 days were identified to be the optimal period to perform a tracheostomy if ventilatory support is still required [18]. Therefore, defining PMV as >10 days seems to be more beneficial to early devise an individualized patient treatment plan.

## Discussion

Predicting prolonged mechanical ventilation (PMV) in patients with TBI is of utmost importance. This importance stems from the fact that early liberation from mechanical ventilation yields improved outcomes [8, 17]. Nevertheless, predicting PMV is proven to be a difficult mission due to several factors such as lack of consensus on the PMV definition and the poor outcomes of the conventional analytical techniques that were used to make predictions. The traditional statistical techniques help clinicians predict PMV by 59% of the time only [8, 14].

Hence, it is very important to help clinicians early identify patients at risk for PMV in order to design an individualized care plan and to decide early tracheostomy to helps patient achieve better outcomes if MV is still required. The lack of a consensual definition of PMV makes the determination of the optimal MV duration and the tracheostomy decision very difficult. We opted for the 10 days to be the cutoff point to differentiate between the PMV and the non-PMV based on a prior Cochrane review which found that 10 days is the optimal duration when moving patients to tracheostomy will result in better outcomes [17]. Therefore, planning early tracheotomy when PMV can be predicted and possibly transferring the patient to the most appropriate institution for PMV and managing its complications is important.

This study demonstrated that the application of supervised machine learning techniques yields moderate overall performance for all the machine learning models in the three sets. Nevertheless, set B achieved more stable performance and higher discrimination power than sets A and C with average AUC 0.813 for the five prediction models compared to average AUC of 0.76 for set A and average AUC of 0.72 for set C. SVM was the chosen prediction technique for set B with accuracy of 0.79 and AUC of 0.84. The importance of this finding is that the optimal prediction performance is achieved when PMV is defined as >10 days which is the optimal period for early tracheostomy [17, 18].

The machine learning techniques in the three sets achieved better performance than the traditional predictive techniques that recorded mild accuracies ranging between 0.60–0.69 and mild AUCs ranging between 0.52 and 0.67. This proves that the machine learning techniques outperform the conventional analytical techniques and can provide more support to the clinicians to make higher quality decisions that improve the patient treatment outcomes.

In set B, the SVM achieved the optimal performance with accuracy of 0.79 and AUC of 0.84. In addition, it performed moderately in the other measures (precision = 0.75, NPV = 0.82, sensitivity = 0.76, specificity = 0.82 and F-score = 0.75). The development of VAP ranked first in predictor importance (0.16). Three quarters of patients who developed VAP ended up with PMV> 10 days compared to 35% of patients who didn’t develop VAP. Ventilator associated pneumonia (VAP) is known to be associated with poor outcomes including PMV and mortality [35]. This finding may not necessarily help early prediction of PMV as VAP is not a condition that a patient presents with when sustains TBI. However, knowing that VAP contributes significantly to the PMV warrants the early implementation of the preventive measures such as VAP bundle of care [36] which is becoming the standard of quality care in critical care medicine.

The need for blood transfusion during resuscitation ranked second in predictor importance with a score of 0.15. Almost 59.8% of patients who received blood for resuscitation needed PMV compared to 19.6% of the patients who didn’t require blood for resuscitation. The need to administer blood transfusion to resuscitate patients who sustain severe trauma could indicate the severity of injury and perhaps a hypovolemic shock that contributes to poor patient`s outcomes. This coincides with Ghiani and colleagues [37] who reported that blood transfusion is independently correlated with worse outcomes. Nevertheless, they concluded that blood transfusion is an indicator for disease severity rather than directly impacting the prognosis. Lai et al. (2013) found that the low hemoglobin level is associated with difficult weaning from MV and may lead to PMV [38]. Also, Zubrow et al. (2018) found that transfusion of Red Blood Cells (RBCs) in pediatrics with acute respiratory distress syndrome is associated with PMV [39].

The GCS at the ED is a significant predictor with predictor importance score of 0.1. In this study, 49.8% of patients who presented to the ED with GCS ≤ 8 stayed on MV longer than 10 days compared to 34.1% of patients who presented to the ED with GCS 13–15 and 26.4% of those who had GCS of 9–12. This finding is consistent with the previous literature which proves that patients with lower GCS are at higher risk for post TBI complications including death [13, 40].

TBI diagnosis (CT scan finding) scored predictor importance of 0.09. 69% of patients who had diffuse axonal injury (DAI) and 66.7% of those who had cerebral edema stayed on mechanical ventilator longer than 10 days. Both cerebral edema and DAI were found to be associated with significant mortality and morbidity [41, 42].

Furthermore, it is found that the greater the HAIS the greater the risk for PMV. Almost 66% of patients who had HAIS = 5 ended with PMV compared to 32% and 45.2% for HAIS 3 and 4 respectively. It is well documented that the severer the TBI, the higher the risk of comorbidities and mortality [25]. The same applies on predicting the PMV[13].

Also, chest AIS scored 0.04 in predictor importance. 58% of patients whose chest AIS 1 and 2 and 52.7% of those whose chest AIS 3–5 had PMV. Previous literature found that chest AIS helps predict the PMV [12]. Okabe found that the severity of blunt chest trauma is significantly associated with the risk of PMV [43].

Sepsis was also found to be among the top ten important predictors for the PMV (predictor importance = 0.04). 83.3% of patients who suffered sepsis following TBI had PMV>10 days. Like VAP, patients may not present to the ED with sepsis right after the TBI. However, knowing that sepsis contributes significantly to the PMV warrants the early implementation of the preventive measures (the 6-hour sepsis bundle) that is a standard of critical care medicine [44].

Furthermore, 62.2% of patients who sustained pneumothorax stayed on MV longer than 10 days. Pneumothorax scored 0.04 in predictor importance. It is evident in the literature that pneumothorax and prolonged chest tube duration are associated with poor outcomes such as PMV and increased ICU length of stay and mortality [45].

In this study, midline shift is ranked number nine (predictor importance 0.04). Midline shift is defined as the “displacement of septum pellucidum in relation to the midline in millimeters” [46]. 52.4% of patients who sustained midline shift had PMV compared to 42.1% of those who didn’t have midline shift. Midline shift is a commonly used variable in predicting post TBI`s unfavorable outcomes (e.g. CRASH and IMPACT tools) [47–49].

The tenth ranked predictor was abdominal AIS (predictor importance 0.03). 59.4% of patients who had abdominal trauma with AIS (1–5) had PMV compared to 41.6% of those who didn’t sustain abdominal injury. Although Blaser et al found that there is a strong correlation between the intra-abdominal hemorrhage and the ICU length of stay and the PMV [50], the severity of the abdominal injury as measured by the AIS was found insignificant predictor of PMV in other studies [12, 13]. This could be attributed to different data processing and inclusion criteria that were followed in the every study.

### Wellbeing and economic values

Predicting PMV is proven beneficial in several aspects. Besides the proven clinical value added, predicting PMV supports the decision of early tracheostomy that is proven economically beneficial. PMV is associated with several complications that contribute to increasing ICU and hospital length of stay that is associated with significantly high cost. Early tracheostomy is associated with reduced ICU and hospital length of stay [17, 51]. It is estimated that the average daily cost of ICU in the USA ranges between $1300 and $9400 depending on the specialization and complexity of patient disease [52]. The daily cost in the ICU increases when mechanical ventilation is required [53]. Therefore, it was found that the early tracheostomy contributes to a significant reduction in the ICU daily cost compared to delayed tracheostomy [19].

Usually, patients’ treatment plans don’t concentrate merely on addressing the acute and the chronic healthcare problems. They focus strongly on enhancing the patient’s quality of life (QOL). Patients who require mechanical ventilation suffer severe deterioration in the QOL [54]. It was found that early liberation from ventilator or early tracheostomy are associated with enhanced QOL [20]. Therefore, this study adds value in several aspects that include clinical, wellbeing and economic aspects.

### Limitations

This study faced several challenges. The size of the sample (674, 643 and 622 records) are considered small for studies that use machine learning techniques. The relatively small sample size has complicated the data processing, partitioning, and model training, validation and testing. Nevertheless, the size of data set that are used and the variables that are included in the study are still comparable with the previous studies. Therefore, the validity of the results shouldn’t be a problem. Also, the data are abstracted from a well validated trauma registry. So, there are several potentially useful predictors that are unobtainable such as laboratory results, ventilator settings, and received medication. Secondly, the lack of agreed upon definition for PMV complicates the classification mission. There are no previous studies that define PMV to be 10 days. This cut-off is determined based on the result that tracheostomy that is done after 10 days of MV is considered late tracheostomy and is associated with unfavourable outcomes. Finally, there are only few studies that aim to predict PMV using machine learning approach. Accordingly, it is difficult to benchmark this study with previous studies that utilize machine learning and to benefit from innovative data processing techniques that are used in prior research. The deployment of the model to support clinical decision making is another significant challenge. This is due to several reasons such as the questionable reliability of the non-traditional predictive techniques that stems to a certain extent from the lack of awareness among the clinicians about the artificial intelligence potentials in supporting clinical decision-making process. Importantly, unlike the logistic regression for instance, the standardized coefficients and the odds ratios pertaining to each predictor in the SVM are not obtainable. This makes the results interpretation more complex than the traditional computational techniques.

## Conclusions

The importance of mechanical ventilation in the critical care settings is increasing due to the increasing demand on the critical care intervention worldwide. Nonetheless, dependence on mechanical ventilation is associated with several serious outcomes. Therefore, the early liberation from mechanical ventilator is of utmost importance. Predicting patients at risk of PMV helps clinicians devise personalized care plans in order to mitigate the risk of PMV and to timely decide tracheostomy if in case ventilatory support is still required. Predicting patients at risk of PMV will not only help improve patients`clinical outcomes, but also helps reduce the critical care cost and enhance patients’ QOL.

The study showed that it is possible to improve the predictive power when using machine learning approach. Nevertheless, more important than making the prediction is to enhance the quality of data in the registry or the electronic medical records which help improve the quality of predictions. Moreover, deploying such models into clinical practice and making them available in a user-friendly way to the clinicians to support their decision-making is of great value.
